# A comparison of patient, intervention, comparison, outcome (PICO) to a new, alternative clinical question framework for search skills, search results, and self-efficacy: a randomized controlled trial

**DOI:** 10.5195/jmla.2020.739

**Published:** 2020-04-01

**Authors:** Lorie A. Kloda, Jill T. Boruff, Alexandre Soares Cavalcante

**Affiliations:** Associate University Librarian, Planning & Community Relations, Library, Concordia University, 1455 boulevard de Maisonneuve O. LB-331.17, Montreal, QC, H3G 1M8, Canada, lorie.kloda@concordia.ca, http://orcid.org/0000-0003-1429-1497; Schulich Library of Physical Sciences, Life Sciences, and Engineering, McGill University, 809 Sherbrooke Street West, Montreal, QC, H3A 0C1, Canada, jill.boruff@mcgill.ca, https://orcid.org/0000-0002-0338-7322; Doctoral (PhD) Candidate, Faculty of Education, McGill University, Montreal, QC, Canada, alexandre.cavalcante@mail.mcgill.ca

## Abstract

**Objective:**

In educating students in the health professions about evidence-based practice, instructors and librarians typically use the patient, intervention, comparison, outcome (PICO) framework for asking clinical questions. A recent study proposed an alternative framework for the rehabilitation professions. The present study investigated the effectiveness of teaching the alternative framework in an educational setting.

**Methods:**

A randomized controlled trial was conducted with students in occupational therapy (OT) and physical therapy (PT) to determine if the alternative framework for asking clinical questions was effective for identifying information needs and searching the literature. Participants were randomly allocated to a control or experimental group to receive ninety minutes of information literacy instruction from a librarian about formulating clinical questions and searching the literature using MEDLINE. The control group received instruction that included the PICO question framework, and the experimental group received instruction that included the alternative framework.

**Results:**

There were no significant differences in search performance or search skills (strategy and clinical question formulation) between the two groups. Both the control and experimental groups demonstrated a modest but significant increase in information literacy self-efficacy after the instruction; however, there was no difference between the two groups.

**Conclusion:**

When taught in an information literacy session, the new, alternative framework is as effective as PICO when assessing OT and PT students’ searching skills. Librarian-led workshops using either question formulation framework led to an increase in information literacy self-efficacy post-instruction.

## INTRODUCTION

In educating students in the health professions about evidence-based practice, instructors and librarians typically use the patient, intervention, comparison, outcome (PICO) framework for asking and classifying clinical questions. This framework, first proposed by Richardson and colleagues [1] along with the categories of therapy, diagnosis, prognosis, and harm/etiology proposed by Sackett and colleagues [2], has been taught to students in occupational therapy (OT) and physical therapy (PT). Though students in the health professions are commonly taught the PICO framework, research to date has been unable to consistently demonstrate that this improves clinical question quality [3], search skills, or search results [4–6]. The present study aimed to determine if a new, alternative clinical question framework was equally or more effective than PICO for improving students’ search skills, search results, and self-efficacy.

Prior research has suggested that converting information needs into structured questions increases the likelihood of information seeking taking place [7]; however, the use of the PICO clinical question structure, even by librarians, has not been shown to result in better search results compared to a more flexible approach [6, 8]. A recent systematic review by Eriksen and Frandsen identified only three research studies investigating the impact of PICO on search performance, none of which demonstrated an effect. Rather, the only clear conclusion was the link between the number of search blocks in the search strategy and the quality of the retrieved search results [6]. A study by Booth and colleagues demonstrated that although a structured search form provided to librarians led to more detailed questions and precise searches, the librarians expressed a preference for a less-structured approach [8].

It appears, therefore, that teaching students to structure their information needs more specifically may be worthwhile for motivating their information seeking and for finding evidence, but it remains unproven whether PICO actually improves the quality of the search results. Because question formulation is the first step in the evidence-based practice cycle, poorly formed questions can have a negative impact on students’ understanding of and success with the rest of the cycle. Though there are at least nine different clinical question frameworks proposed in the literature along with PICO [9, 10], these remain theoretical and have yet to be tested for their effectiveness.

A recent study with clinicians in OT and PT has proposed a clinical question framework based on their everyday information needs that have been identified in the clinical context [11]. This new, alternative framework has eight elements: problem, intervention, population, outcome measure, time, context, professional stakeholder, and patient or family stakeholder (Table 1). Unlike in the PICO framework where only the *comparison* element is optional, in the alternative question framework all of the elements are optional and can be used in any combination.

**Table 1:** Structural elements in clinical questions using the alternative framework

Element	Definition
Problem	Describes the condition or situation of interest to the therapist that requires an intervention, assessment, or more information of any kind.
Population	Describes the patient population or client group. May be demographic in nature or specify a health condition.
Intervention	Describes a treatment (preventative or therapeutic), an assessment, or diagnostic tool, or some other type of service or condition to which a patient might be exposed.
Context	Describes the setting or location of the patient or intervention. May include a health care or a community setting.
Temporality (Time)	Specifies a time period or sequence relating to any other element, such as the duration of an intervention, disease stage, or points in time at which the outcome is measured.
Professional stakeholder	Describes the point of view of one or more types of health professionals.
Patient or family stakeholder	Identifies the patient and family members as individuals with a vested interest in the answer or the outcome of that answer.
Outcome measure	Specifies a measurable result, whether for impact of treatment or normal values for an assessment tool.

Given that this alternative framework was derived from research on clinicians’ information needs in their everyday practice, the authors asked whether this alternative framework would prove to be equally or more effective than the standard PICO framework in teaching future clinicians in OT and PT to seek out and use evidence in their practice. The study’s research questions were:

1. Do students in OT and PT who are taught information literacy skills using the alternative clinical question framework conduct literature searches with similar search results as those taught the PICO framework?
2. Do students in OT and PT who are taught the alternative clinical question framework demonstrate similar search skills as those taught the PICO framework?
3. Do students in OT and PT who are taught the alternative clinical question framework show a difference in information literacy self-efficacy when compared to those taught the PICO framework?
4. What are the perceptions and experiences of students with regard to clinical question frameworks?

## METHODS

**Figure 1:**
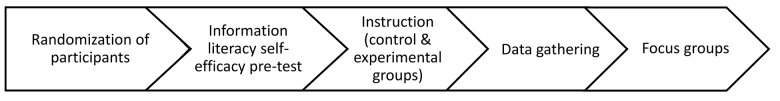
Study timeline

To answer the research questions, we conducted a randomized controlled trial. An overview of the study timeline is provided in Figure 1.

### Population and sample

All OT and PT students enrolled in a required credit-bearing course that typically includes advanced information literacy instruction in their respective programs were invited to participate. The population was composed of 75 OT students and 76 PT students, for a total of 151 students. These students were in either their final year of study toward their undergraduate degrees before a direct-entry master’s or in their qualifying year preceding the master’s degree program.

### Setting

The School of Physical and Occupational Therapy at McGill University has a robust program for students that incorporates evidence-based practice learning outcomes throughout the curriculum. The school’s instructors design, deliver, and assess content on evidence-based practice. Librarians also conduct information literacy instruction and assessment at strategic points throughout the curriculum, and in many instances learning outcomes of that instruction directly support evidence-based practice outcomes [12].

For this study’s population of interest, library instruction takes place in September for OT students during the “Therapeutic Strategies” course and for PT students as part of the “Neurological Rehabilitation” course. Up until the time of this study, information literacy learning outcomes were included in the course syllabi for both of these courses, and the liaison librarian for rehabilitation sciences was responsible for teaching this content. The library instruction session, a required component of the students’ courses, had already been designed to address the following learning outcomes:

1. to identify an information need from a clinical case or patient scenario and translate this into a clinical question
2. to select appropriate sources for locating evidence
3. to effectively search the MEDLINE database (OvidSP platform) for records to answer a clinical question using advanced search strategies

### Randomization

Participants were randomly allocated to the experimental group (the alternative clinical question framework) or the control group (the PICO framework) using an online random number generator after consent was obtained. The allocation was concealed from the students until the instruction session during class time. Four instruction sessions were held: two for OT students (control and experimental) and two for PT students (control and experimental). Students who did not consent to participate in the study received instruction with the control group.

### Instruction

Both the control and experimental groups were taught by the same librarian instructor who was also one of the researchers (Boruff), for the same ninety-minute duration, and in a face-to-face setting with identical teaching methods (i.e., a combination of lecture and hands-on activities) in a computer-lab classroom setting. Some of the examples used in the teaching were tailored specifically for OT students and PT students. The learning content of the session was based on previous years, and the presentation of the material was redesigned with the assistance of a graduate student in education with experience in educational design (Cavalcante). The slide decks used for the instructional session including content for both control and experimental groups are available online from SlideShare: Searching with PICO and Alternative question formulation framework OT and SlideShare: Searching with PICO and Alternative question formulation framework--Physical Therapy.

Participants in both the control and experimental groups learned about the categories of question types (therapy, diagnosis, prognosis, and harm/etiology), selection of information resources based on these categories, and advanced search skills in the MEDLINE database (OvidSP platform). Participants in the control group learned about the PICO framework and used it to determine the search concepts for two example scenarios. Participants in the experimental group learned about the alternative framework and used it to determine search concepts for two example scenarios. The difference between the two sessions amounted to three slides. Supplemental Appendix A provides an example scenario and how each framework could be applied to the scenario.

### Data gathering

At the outset of each instruction session, participants completed a demographic questionnaire and a short instrument measuring their information literacy self-efficacy (i.e., pre-test). This twelve-item instrument was based on a longer, validated information literacy self-efficacy instrument [13].

Three weeks after the experimental and control groups received their instruction sessions, a data-gathering session was held for all participants. During this one-hour session held in a computer classroom, participants received a clinical scenario and were asked to respond to three questions about the scenario: one to document the concepts or elements in the scenario, another to document a clinical question arising from the scenario, and the third to document their MEDLINE search strategies and the records that they identified as relevant. The clinical scenario that was provided was identical for all participants and was designed in consultation with content experts to be relevant for both OT and PT (supplemental Appendix B). All responses were submitted electronically using the LimeSurvey tool. During this session, participants once again completed the instrument for measuring their information literacy self-efficacy (i.e., post-test).

None of the information gathered was shared with course instructors or used as part of students’ course assessments. All participants’ identities were masked and coded so that scores for the information literacy self-efficacy instrument could be compared pre- and post-instruction. After the data gathering sessions were completed, all OT and PT students were provided with an electronic copy of the slides with content for both the experimental and control groups for their own information. The students were also offered the opportunity to attend a workshop that covered the material that was not covered in the session they attended (i.e., the experimental group could receive the control group instruction and vice versa). No participants elected to attend.

Near the end of the term, focus groups were held with participants from both the control and experimental groups. The focus group interviews were held to gather information about students’ perceptions and experiences of learning about and using the frameworks and were facilitated by one of the researchers (Kloda). The timing of the focus group session was chosen to allow participants time to complete their coursework and reflect on the learning outcomes from the workshop. By the end of the term, participants were familiar with both the PICO framework and the alternative framework. Issues addressed in the focus groups included how the framework helped students identify their information needs, select sources, and search for answers; how the frameworks influenced their confidence in engaging in information seeking; and the perceived utility of using a clinical question framework in OT and PT. Focus group interviews were not recorded. Rather, field notes were taken by both the focus group facilitator (Kloda) and research assistant (Cavalcante) and compared afterward.

### Ethics, consent, benefits, and harms

Ethical approval was obtained from the institutional review boards of the universities of both lead investigators, and the study received support from the course coordinators and the program directors at the School of Physical and Occupational Therapy. All students who consented to participate in the study were provided with an incentive gift card worth $20 immediately following the study data-gathering session that they attended.

### Outcome measure

The primary outcome was the accuracy of the search results for the searches performed by participants to answer the questions arising from the clinical scenario. Accuracy was calculated using the F-measure [14], which in itself is determined by two related measures:

- Recall (also called sensitivity) is the ratio of the number of relevant records retrieved by the search strategy, compared to the total number of relevant records (i.e., gold standard reference set) in the database as identified by content experts. The ideal recall ratio is 1 or 100%.
- Precision is the ratio of the number of relevant records retrieved compared to the total number of records retrieved. A ratio of 1 is nearly impossible, but higher values are better. The “number needed to read” is calculated by dividing 1 by the precision value (e.g., for a search result set with a precision of 0.2, the number needed to read is 5, meaning one would have to “read” or look over 5 records to find 1 relevant record).

The F-measure is the weighted harmonic mean of recall and precision, with recall and precision equally weighted:

$$2\times \frac{\text{precision}\times \text{recall}}{\text{precision}+\text{recall}}$$

Search performance was based on a comparison of the results of participants’ literature searches that were conducted with respect to a clinical scenario applicable to both OT and PT students. Each participant’s search strategy and results were compared to the gold standard reference set as evaluated by content experts (instructors in OT and PT). Recall, precision, and the F-measure were calculated for each participant’s search result set in both the control and experimental groups.

The secondary outcomes were participants’ search skills, information literacy self-efficacy, and perceptions of the clinical question formulations for structuring their information seeking.

Search skills in the context of evidence-based practice were measured using participants’ stated concepts, clinical questions, and search strategies that they conducted in MEDLINE. The original intention was to use the clinical scenarios as well as questions one and four from the Adapted Fresno Test [15] (validated for use with OTs) and Modified Fresno Test [16] (validated for use with PTs), which are the best options for assessing these skills for rehabilitation students [17]. However, there were two problems with these tests. First, the content experts determined that the subjects of the scenarios were outdated or inappropriate for the student population. Second, question four from the Adapted and Modified Fresno Tests does not test the skills of an actual search but rather asks for a description of how one would search.

Due to these constraints, the rubric for question formulation was used from the Modified Fresno Test (with the point scheme furthered modified) (supplemental Appendix C), and the research team created its own rubric to assess the search strategy (supplemental Appendix D). The content experts assisted the research team in writing a clinical scenario that was relevant to both OTs and PTs. A librarian (blinded to the participants’ membership in the control or experimental group and distinct from the research team) used a gold standard to score the clinical questions out of a possible sixteen points and search strategies out of a possible ten points using the rubrics mentioned above. The gold standard search strategy and search results were created by one of the investigators (Boruff) and validated by another (Kloda), both of whom are expert searchers. The lists of articles that were retrieved were given to content experts to choose the most relevant results for the purposes of calculating the F-measure. The research team verified that no new results were retrieved by the gold standard search on the days of data collection.

Twelve items were selected and adapted from the information literacy self-efficacy scale [13] that were relevant to the skills of defining information needs, selecting sources, and developing and executing search strategies. For each item, participants rated their own skills on a seven-point Likert scale (1=almost never true, 7=almost always true). This instrument was administered pre- and post-instruction to both experimental and control groups to determine if the instruction led to an improvement in self-efficacy and to determine any differences between control and experimental groups. The twelve items in the scale are listed in supplemental Appendix B along with the clinical scenario.

### Data analysis

SPSS, version 24, was used to calculate descriptive statistics (e.g., means and standard deviations) and perform inferential tests to compare search performance and pre- and post-instruction scores from the information literacy self-efficacy questionnaire between groups.

To understand participants’ perceptions of the clinical question frameworks, qualitative analysis was undertaken of the field notes taken by two individuals present during the focus group, neither of which was the librarian who provided the instruction, and themes were identified. All members of the research team read through all the field notes and discussed the various responses to questions and points made during the discussion to ensure that all points of view were included in the results.

## RESULTS

### Participants

**Figure 2:**
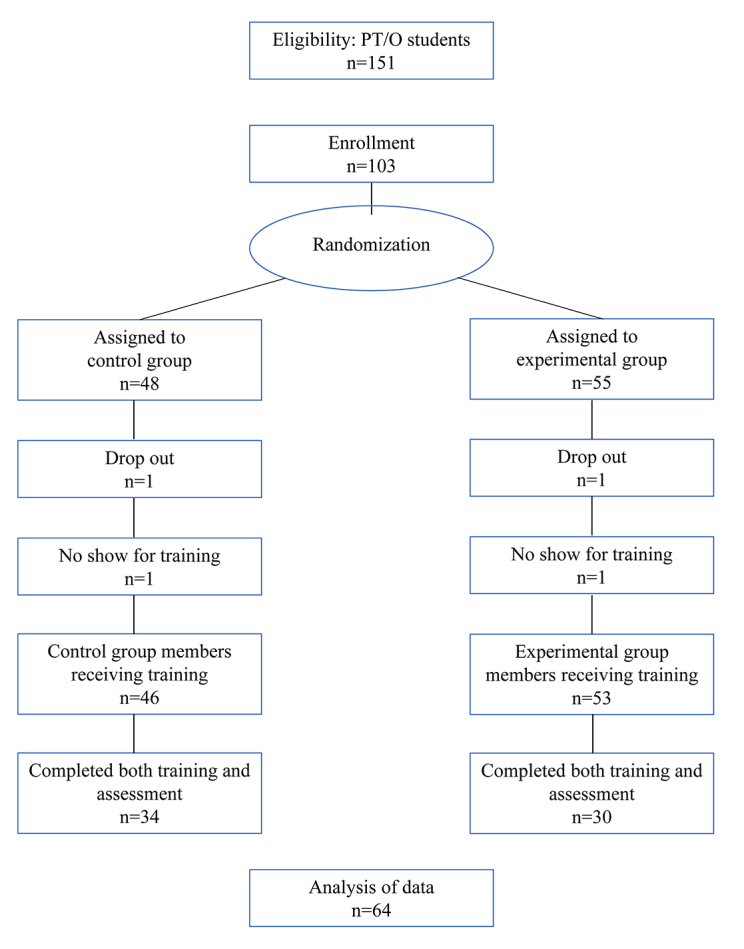
Study participants

Out of a possible 151 eligible OT and PT students, 103 consented to participate in the study, but several were lost due to withdrawal, drop-out, and failure to follow-up, leaving 64 with data. Of those assigned to the control group (PICO), 34 completed both the training and assessment instruments. Of those assigned to the experimental group (alternative framework), 30 completed both the training and assessment (Figure 2).

### Search performance

Independent samples *t*-tests were conducted to determine if there was a difference in search performance between the control and experimental groups. There were no significant differences in recall (*p*=0.167), precision (*p*=0.243), or the F-measure (*p*=0.163) (Table 2).

**Table 2:** Search performance

	Group	n	Mean	Standard deviation
Recall	Control	34	0.17059	0.270274
	Experimental	30	0.27333	0.317244
Precision	Control	34	0.10136	0.177778
	Experimental	30	0.15476	0.184262
F-measure	Control	34	0.11600	0.184657
	Experimental	30	0.18478	0.204710

### Search skills

For the secondary outcome of search skills, 3 scores were calculated for each participant. The clinical question was scored out of a possible 16 points. The search strategy was scored out of a possible 10 points, and the number of concepts was counted. Out of 64 participants who completed the data gathering activity, 43 provided their search strategy (21 in the control group and 22 in the experimental group). Independent samples *t*-tests showed no significant difference in points for the clinical question (*p=*0.653), search strategy (*p*=0.676), or the number of questions concepts identified (*p*=0.178) (Table 3).

**Table 3:** Search skills

	Group	n	Mean	Standard deviation
# of concepts (open-ended)	Control	34	3.53	1.107
	Experimental	30	3.90	1.062
Clinical question (/16)	Control	21	9.10	2.809
	Experimental	22	8.73	2.511
Search strategy (/10)	Control	21	6.14	3.198
	Experimental	22	6.50	2.304

### Information literacy self-efficacy

A total information literacy self-efficacy score was calculated for each participant by summarizing the values (on 7-point Likert scales) for all 12 items. The lowest possible score was 12, and the highest was 84. Wilcoxon rank sum tests showed no significant differences between the control and experimental groups in self-efficacy scores at pre-instruction (*p*=0.941) and post-instruction (*p*=0.772). However, a paired *t*-test showed that post-instruction self-efficacy scores were significantly higher than pre-instruction scores across all participants (*t*(63)=2.627, *p*=0.001) (Table 4).

**Table 4:** Information literacy self-efficacy pre- and post-instruction

	n	Mean	Standard deviation	Minimum score	Maximum score	Group	n	Mean rank
Pre-instruction	64	63.41	10.00	39	84	Control	34	32.66
						Experimental	30	32.32
Post-instruction	64	66.56	7.96	46	82	Control	34	33.13
						Experimental	30	31.78

### Perceptions of the two frameworks

Focus groups were held with two groups of participants, one with OT students and one with PT students. Both groups consisted of a combination of participants from the control and experimental groups. A total of eleven participants attended the focus group discussions. The participants did not express a strong preference for either PICO or the alternative framework for articulating the clinical question. Advantages and drawbacks were identified for each. For example, in one instance, a participant who was already familiar with PICO from a prior course (and who was in the experimental group in the present study) was drawn to using the PICO question structure out of habit. The most critical factor expressed by the focus group participants in the type of clinical questions structure was consistency. The OT and PT programs included several courses in which evidence-based practice concepts were taught, and participants preferred a consistent approach to the clinical questions structure, whichever was selected.

## DISCUSSION

Our finding that the alternative framework was as effective as PICO in teaching OT and PT students how to search for evidence to answer their clinical questions reinforces results of prior research, which do not demonstrate clear improvements in search skills when teaching specific types of clinical question frameworks over others [3–6]. A possible reason for this finding is that the specific clinical question framework is not the determining factor in improving students’ and clinicians’ ability to conduct effective literature searches. Rather, it is the instruction, including learning outcomes of clearly identifying the information need using a clinical question, that influences the success of the literature search and the individual’s self-efficacy.

There are benefits to using the alternative framework in information literacy workshops. The instruction librarian (Boruff) found that teaching searching skills to OT and PT students using the alternative framework made it easier to explain why some concepts in a scenario are included as search terms and other concepts are better served as limits or details to look for in the reference. The alternative framework seemed to help students think more flexibly about search terms and gave the librarian a tool to explain complex scenarios, compared to using the PICO framework. Following this positive experience and considering the comments from the focus groups about consistency, the instruction librarian is working with the School of Physical and Occupational Therapy to investigate the possibility of introducing the alternative framework into the curriculum earlier so that students are familiar with both frameworks throughout their studies.

Participants’ information literacy self-efficacy scores increased after instruction across both the experimental and control groups, further demonstrating that either the alternative framework or the PICO framework was effective for increasing information literacy self-efficacy. Participants’ scores were already high pre-instruction, which might be attributed to previous education and experience or to false perceptions. A more accurate measure would be to assess participants’ information literacy skills using a tool validated with this population, such as the Open Test of Information Literacy [18].

We expected the number of concepts identified to be higher for the experimental group due to the higher number of possible elements in the alternative framework. Though the number of concepts that study participants identified was marginally higher for the experimental group, the difference between groups was not statistically significant. There are several possible explanations for this result. First, participants were not provided with the PICO and alternative framework elements during the data gathering session and, therefore, would not necessarily have remembered the one they were taught. The alternative framework, having twice the number of possible elements (eight) compared to PICO (four), would be much harder to remember. Second, the number of relevant concepts identified by participants might have simply been a function of the scenario itself not having many elements to identify. Third, having been taught question formulation as a precursor to conducting a database search, participants might have been preparing for a search with three or four search terms to be combined and stopped after having identified that many.

This study has limitations stemming from the difficulty in eliminating the bias toward the PICO framework. Although every effort was made to keep participants in both control and experimental groups unaware of the content of the information literacy workshop of the other group, it was likely that some had learned about the PICO clinical question framework earlier in their studies. This meant that some of the participants in the experimental group would have been aware of PICO in addition to being taught the alternative framework and might have been biased in favor of the PICO framework as a result. Additionally, in an attempt to use a previously validated measure—the published Adapted and Modified Fresno Test rubrics for scoring clinical questions, which are based on the PICO framework—might have biased scores toward clinical questions that incorporated the four elements of PICO. Though we could have further adapted the Fresno Test for the alternative clinical question framework, its widespread use as a validated tool made it ideal as a benchmark for comparison.

Future research could explore the effect of having students and health professionals convert their information needs into clinical questions using a framework on their subsequent information-seeking behavior. This information-seeking behavior could include the number and type of sources consulted, as well as time spent engaged in information seeking, rather than being limited to database search strategies and results.

This is the first study to investigate the effectiveness of teaching information literacy skills to health professional students using the new, alternative clinical question framework. The results demonstrate that providing librarian-led instruction using the alternative framework is just as effective as using the PICO framework. Considering the flexibility that the alternative framework allows students when designing a search strategy, librarians teaching information literacy sessions in evidence-based practice contexts may want to use the alternative framework in their own workshops. Teaching OT and PT students using the alternative framework is not only feasible, but also an effective way to teach information literacy that incorporates question formulation based on real-world clinical experience in the rehabilitation professions.

## SUPPLEMENTAL FILES

Appendix A Question formulation example using each framework

Appendix B Search skills activity

Appendix C Question formulation rubric for comparison of patient, intervention, comparison, outcome (PICO) to new framework (taken from Modified Fresno Test; point scheme modified)

Appendix D Search skills grading rubric for comparison of patient, intervention, comparison, outcome (PICO) to new framework

## 

**Lorie A. Kloda, AHIP**, lorie.kloda@concordia.ca, http://orcid.org/0000-0003-1429-1497, Associate University Librarian, Planning & Community Relations, Library, Concordia University, 1455 boulevard de Maisonneuve O. LB-331.17, Montreal, QC, H3G 1M8, Canada

**Jill T. Boruff, AHIP**, jill.boruff@mcgill.ca, https://orcid.org/0000-0002-0338-7322, Schulich Library of Physical Sciences, Life Sciences, and Engineering, McGill University, 809 Sherbrooke Street West, Montreal, QC, H3A 0C1, Canada

**Alexandre Soares Cavalcante**, alexandre.cavalcante@mail.mcgill.ca, Doctoral (PhD) Candidate, Faculty of Education, McGill University, Montreal, QC, Canada
