# Supplementary material for: A comparison of patient, intervention, comparison, outcome (PICO) to a new, alternative clinical question framework for search skills, search results, and self-efficacy: a randomized controlled trial
Source: J Med Libr Assoc. 2020 Apr 1;108(2):185–94. doi: 10.5195/jmla.2020.739 (PMC7069809; doi:10.5195/jmla.2020.739)
Supplement: Appendix A [file jmla-108-185-s001.pdf]

JMLA Journal of the Medical Library Association

SC

Supplemental content to
J Med Libr Assoc. Apr;108(2):dx.doi.org/10.5195/jmla.2020.739
www.jmla.mlanet.org
© Kloda, Boruff, Soares Cavalcante 2020

A comparison of patient, intervention, comparison, outcome (PICO) to a new, alternative clinical question framework for search skills, search results, and self-efficacy: a randomized controlled trial

Lorie A. Kloda, AHIP; Jill T. Boruff, AHIP; Alexandre Soares Cavalcante

APPENDIX A

Question formulation example using each framework

Mrs. Holiday is a sixty-five-year-old female who is living with the effects of a stroke that happened two years ago. Her recovery has allowed her to get back to work and return to leisure activities, albeit in a modified manner. Her stroke was in her left middle cerebral artery and was considered moderate. She is consulting you, the physical therapist, because for the last two months she is having trouble completing her weekly hikes due to difficulty controlling her right leg during the walks. You have determined one of the main contributing factors to this problem is the presence of spasticity in the right leg. You know that transcutaneous electric nerve stimulation is used to address this problem, but you wonder if a combined modality therapy might be more effective for Mrs. Holiday in order to improve her ability to control her right leg during walks.

Patient, intervention, comparison, outcome (PICO) framework

Patient	65-year-old female stroke survivor with spasticity in right leg
Intervention	Combined modality therapy
Comparison	Transcutaneous electric nerve stimulation alone
Outcome	Improved motor function
Type of question	Therapy
Answerable clinical question	In stroke survivors with lower limb spasticity, is a combined modality therapy more effective than transcutaneous electric nerve stimulation alone to improve motor function?

JMLA Journal of the Medical Library Association

SC
Supplemental content to
J Med Libr Assoc. Apr;108(2):dx.doi.org/10.5195/jmla.2020.739
www.jmla.mlanet.org
© Kloda, Boruff, Soares Cavalcante 2020

Alternative framework

Problem	Spasticity in right leg
Population	65-year-old female
Patient stakeholder	
Professional stakeholder	Physical therapist
Intervention	Transcutaneous electric nerve stimulation, combined modality therapy
Context	Leisure activities
Outcome measure	Improved motor function
Time	Stroke 2 years ago
Type of question	Therapy
Answerable clinical question	In stroke survivors with lower limb spasticity, is a combined modality therapy more effective than transcutaneous electric nerve stimulation alone to improve motor function?
