# Supplementary material for: A comparison of patient, intervention, comparison, outcome (PICO) to a new, alternative clinical question framework for search skills, search results, and self-efficacy: a randomized controlled trial
Source: J Med Libr Assoc. 2020 Apr 1;108(2):185–94. doi: 10.5195/jmla.2020.739 (PMC7069809; doi:10.5195/jmla.2020.739)
Supplement: Appendix B [file jmla-108-185-s002.pdf]

JMLA Journal of the Medical Library Association

Supplemental content to
J Med Libr Assoc. Apr;108(2):dx.doi.org/10.5195/jmla.2020.739
www.jmla.mlanet.org
© Kloda, Boruff, Soares Cavalcante 2020

A comparison of patient, intervention, comparison, outcome (PICO) to a new, alternative clinical question framework for search skills, search results, and self-efficacy: a randomized controlled trial

Lorie A. Kloda, AHIP; Jill T. Boruff, AHIP; Alexandre Soares Cavalcante

APPENDIX B

Search skills activity

Please read the clinical scenario below and try to answer all of the following questions to the best of your ability.

Mrs. Smith is a thirty-eight-year-old female who has been referred to you for low back pain following an injury at work. She is employed as a plumber. You plan on developing an exercise therapy program for her, and you wonder if Pilates would be good to add to her program.

1. Use this box to write down the main elements or ideas in this scenario.

2. Write a focused clinical question for this scenario to help you organize a search of the clinical literature.

3. Go into Ovid MEDLINE (use this link: [direct link to Ovid MEDLINE]) and conduct a search to find articles that help answer your focused clinical question.

Select all relevant articles from your search results list (those that answer your clinical question).

In the box below, paste your complete MEDLINE search strategy and the citations of the relevant articles you have selected (the abstract is not necessary).

Please follow the instructions provided in the handout to copy and paste these items from Ovid (if you have technical difficulties with the copy and pasting, you may ask the study moderator for help).

JMLA Journal of the Medical Library Association

SC
Supplemental content to
J Med Libr Assoc. Apr;108(2):dx.doi.org/10.5195/jmla.2020.739
www.jmla.mlanet.org
© Kloda, Boruff, Soares Cavalcante 2020

Information literacy self-efficacy scale

"I feel that I am able to…"

1. Define the information I need
2. Identify a variety of potential sources of information
3. Limit search strategies by subject, language, and date
4. Conduct search strategies using subject headings
5. Conduct search strategies using keywords
6. Conduct search strategies using a combination of subject headings and keywords
7. Conduct subject headings using logical operators (AND, OR)
8. Decide where and how to find the information I need
9. Use electronic information sources
10. Locate information sources in the library
11. Use several resources to conduct a search
12. Select information to respond to the information need
