# Supplementary material for: A comparison of patient, intervention, comparison, outcome (PICO) to a new, alternative clinical question framework for search skills, search results, and self-efficacy: a randomized controlled trial
Source: J Med Libr Assoc. 2020 Apr 1;108(2):185–94. doi: 10.5195/jmla.2020.739 (PMC7069809; doi:10.5195/jmla.2020.739)
Supplement: Appendix C [file jmla-108-185-s003.pdf]

JMLA Journal of the Medical Library Association

Supplemental content to
J Med Libr Assoc. Apr;108(2):dx.doi.org/10.5195/jmla.2020.739
www.jmla.mlanet.org
© Kloda, Boruff, Soares Cavalcante 2020

A comparison of patient, intervention, comparison, outcome (PICO) to a new, alternative clinical question framework for search skills, search results, and self-efficacy: a randomized controlled trial

Lorie A. Kloda, AHIP; Jill T. Boruff, AHIP; Alexandre Soares Cavalcante

APPENDIX C

Question formulation rubric for comparison of patient, intervention, comparison, outcome (PICO) to new framework (taken from Modified Fresno Test; point scheme modified)

QUESTION #1: Read the clinical scenario. Write a focused clinical question for that scenario that will help you organize a search of the clinical literature.

	Excellent (4)	Strong (3)	Limited (2)	Minimal	Not evident (0)
a: Population<br>/4	Multiple relevant descriptors; e.g., “work-related injury,” “female,” or “acute,” or “low-back pain”; e.g., “boy with hemiparesis” specific age group, gender, diagnosis, motor presentation	One appropriate descriptor as above examples; e.g., “women,” or “worker,” or “low-back pain”; e.g., “hemiparesis,” “boy,” “10-year-old,” “post-stroke”	A single general descriptor unlikely to contribute to search; e.g., “patient”		None of the above present

JMLA Journal of the Medical Library Association

Supplemental content to
J Med Libr Assoc. Apr;108(2):dx.doi.org/10.5195/jmla.2020.739
www.jmla.mlanet.org
© Kloda, Boruff, Soares Cavalcante 2020

	Excellent (4)	Strong (3)	Limited (2)	Minimal	Not evident (0)
b: Intervention<br>/4	Includes specific intervention of interest (intervention could be a diagnostic technique):<br>• manual therapy<br>• specific individual components of manual therapy<br>• combination of exercise and manual therapy<br>• task-specific strengthening		Mentions intervention but unlikely to contribute to search; e.g., "methods," "options," "treatments"		None of the above present
c: Comparison<br>/4	Identifies specific alternative of interest; e.g., "no manual therapy"; "low intensity stretching"		Mentions comparison but unlikely to contribute to search; e.g., "alternate methods"		None of the above present
d: Outcome<br>/4	Outcome that is objective and meaningful to patient or patient case (if question is diagnostic, should relate to diagnosis trying to detect); e.g., return to work, pain reduction, injury prevention; e.g., selective motor control or functional use of paretic extremities, walking velocity	Nonspecific outcome:<br>• recovery<br>• spasticity<br>• tone<br>• strength	Reference to outcome, but so general as to be unlikely to contribute to search:<br>• effects<br>• change the outcome<br>• effective<br>• improvement<br>• success<br>• change the outcome		None of the above present
