# Supplementary material for: A comparison of patient, intervention, comparison, outcome (PICO) to a new, alternative clinical question framework for search skills, search results, and self-efficacy: a randomized controlled trial
Source: J Med Libr Assoc. 2020 Apr 1;108(2):185–94. doi: 10.5195/jmla.2020.739 (PMC7069809; doi:10.5195/jmla.2020.739)
Supplement: Appendix D [file jmla-108-185-s004.pdf]

Journal of the Medical Library Association

Supplemental content to
J Med Libr Assoc. Apr;108(2):dx.doi.org/10.5195/jmla.2020.739
www.jmla.mlanet.org
© Kloda, Boruff, Soares Cavalcante 2020

A comparison of patient, intervention, comparison, outcome (PICO) to a new, alternative clinical question framework for search skills, search results, and self-efficacy: a randomized controlled trial

Lorie A. Kloda, AHIP; Jill T. Boruff, AHIP; Alexandre Soares Cavalcante

APPENDIX D

Search skills grading rubric for comparison of patient, intervention, comparison, outcome (PICO) to new framework

Points/10	(4)	(3)	(2)	(1)	Incorrect/Missing (0)
Use of Medical Subject Headings (MeSH)<br><br>/4	Selects all appropriate MeSH terms	Excludes one MeSH term<br><br>Example: "low blood pressure" instead of Hypertension/<br><br>OR<br><br>Uses an incorrect or inappropriate MeSH term, subheading or limit one time.<br><br>Examples:<br>• Uses Adult/ for age limit<br>• Limits to "middle age"<br>• Uses Blood Pressure/ instead of Hypertension/<br>• Selects irrelevant MeSH in addition to relevant MeSH	Excludes two MeSH terms<br><br>OR<br><br>Uses two incorrect or inappropriate MeSH terms, subheadings, or limits<br><br>See examples to the left	Excludes more than two MeSH terms<br><br>OR<br><br>Uses multiple incorrect or inappropriate MeSH terms, subheadings, or limits	Fails to use any MeSH terms<br><br>OR<br><br>Fails to provide search history

JMLA Journal of the Medical Library Association

SC
Supplemental content to
J Med Libr Assoc. Apr;108(2):dx.doi.org/10.5195/jmla.2020.739
www.jmla.mlanet.org
© Kloda, Boruff, Soares Cavalcante 2020

Points/10	(4)	(3)	(2)	(1)	Incorrect/Missing (0)
Use of keywords<br>/2			Uses keywords when necessary for search (if keywords not necessary, and they don't use them at all or use them in conjunction with MeSH, give full points).<br>Example: "Pilates" with subject heading Exercise Movement Techniques/	Uses keywords inappropriately<br>Example: Makes the keywords too specific	Fails to use keyword when necessary for the search
Use of Boolean logic<br>/4	Uses AND or OR to combine concepts appropriately	Uses Boolean operator incorrectly one time.<br>Examples:<br>Uses OR between two concepts instead of AND<br>Uses AND between two concepts instead of OR	Uses Boolean operator incorrectly two times		Repeatedly uses Boolean operators incorrectly<br>OR<br>Does not provide search history
